# Age Estimation in 0–8-Year-Old Children in France: Comparison of One Skeletal and Five Dental Methods

**DOI:** 10.3390/diagnostics13061042

**Published:** 2023-03-09

**Authors:** Laurent Martrille, Stavroula Papadodima, Cristina Venegoni, Nicolas Molinari, Daniele Gibelli, Eric Baccino, Cristina Cattaneo

**Affiliations:** 1 EDPFM, University of Montpellier, Department of Legal Medicine, CHU Montpellier, F-34000 Montpellier, France; 2 Department of Forensic Medicine and Toxicology, School of Medicine, National and Kapodistrian University of Athens, M. Asias 75, 115 27 Athens, Greece; 3 LABANOF, Laboratorio di Antropologia e Odontologia Forense, Sezione di Medicina Legale, DMU—Dipartimento di Morfologia Umana e Scienze Biomediche, Università degli Studi di Milano, Via Mangiagalli 37, 20133 Milano, Italy; 4 IDESP, INSERM, PreMEdical INRIA, University of Montpellier, CHU Montpellier, F-34000 Montpellier, France

**Keywords:** forensic anthropology, age estimation, skeletal growth, dental development, children, pediatric population

## Abstract

Age estimation in juveniles is a critical procedure in judicial cases for verification of imputability or for civil reasons when adopting children. Several methods based both on skeletal and dental growth have been performed and applied on different populations; nevertheless, few articles have compared different methods in order to test their reliability in different conditions and age ranges, and this is a clear obstacle in the creation of common guidelines for age estimation in the living. A comparison of five dental methods (Anderson, Ubelaker, Schour and Massler, Gustafson and Koch, Demirjian) and one skeletal method (Greulich a Pyle atlas) was performed on a population of 94 children aged between 0 and 8 years. Results showed that, whereas under 2 years all the methods have the same inaccuracy, over 2 years the diagram methods, such as Schour and Massler and Ubelaker’s revised one, have a lower error range than the most frequently used Greulich and Pyle atlas and Demirjian method. Schour and Massler, Gustafson and Koch, and Ubelaker methods showed, respectively, a mean error amounting to 0.40, 0.53, and 0.56 years versus the 0.74 and 0.88 years given by Demirjian and the Greulich and Pyle atlas. An in-depth analysis of the potential of several methods is necessary in order to reach a higher adherence of age estimation with the complexity of growth dynamics.

## 1. Introduction

Age estimation is a crucial procedure both in the living and in cadavers, with different aims; in the case of unidentified decedents, age estimation is included within the reconstruction of the biological profile (with a diagnosis of sex, ancenstry, and height estimation) [1]. In archaeology, age estimation can give important information about past populations. In living minors, age estimation is usually requested by the judge in order to ascertain imputability and for civil reasons concerning the adoption of children from foreign countries and the need to verify their real age for their insertion in compulsory schooling [1,2] or in their participation in competitive sports [3]. Other age-disputed cases include child labor, arranged marriages, sex trade, and human trafficking [4]. Age estimation is also necessary in cases of unaccompanied minors with unknown ages in the context of asylum procedures and migration [4]. It is worth mentioning that in 2021, European countries recorded 648,000 asylum requests for international protection [5], highlighting the growing need for age estimation in living subadults.

Not only the aims, but also the methods and specific procedures differ between unidentified bodies and the living; the need for non-invasive diagnostic methods prevents one from using age estimation methods commonly applied to cadavers. In addition, the aims of age estimation in the living require a more limited error range (standard deviation) and higher precision; these limits have brought on the development of numerous methods of age estimation, based on the growth of the skeletal district and of the odontological profile taking place in the juvenile. It is obvious that given the risks which may derive from an incorrect age estimation in case of the living, the formulation of guidelines concerning which methods should be used in different situations, and, above all, how to collect results from different methods in order to give a unique result is very important. The first indication concerning the issue of age estimation in the living was given by *The Study group on Forensic Age Diagnostic*, which provided a first indication concerning the general procedure for assessing age in the living, including a clinical examination, X-ray examination of the left hand and wrist, and an dental examination that evaluates an orthopantomogram, followed eventually by CT scan of the clavicle sternal end [6]. The following year, the FASE (Forensic Anthropology Society of Europe) subsection of IALM (International Academy of Legal Medicine) published a review concerning the mean error and field of application of each method and suggested a protocol for the age estimation in the dead and the living [1]. These attempts at defining a common procedure for age estimation highlighted the need for putting an order within the different methods, verifying their performances within different age ranges according to the sex and specific conditions of the single case. This process will lead to a higher individualization of the age estimation procedure, and therefore to a higher precision of the final result; the path to a common way of assessing age is based on the comparison of different methods in order to verify which is the best one and to determine their correlation with real age.

In the past, a comparison between odontological and skeletal methods mainly concerned population affected by infective or growth disease in order to confirm a delayed development in the affected children [7,8]. Several studies have also addressed the comparison of skeletal and odontological methods in populations without specific problems in order to establish the gold standard [2,9,10,11,12].

This study aims at verifying the precision and inaccuracy of five odontological methods (Anderson, Demirjian, Schour and Massler, Ubelaker revised method, Gustafson and Koch) [13,14,15,16,17] and one skeletal—the application of the Greulich and Pyle atlas on children aged between 0 and 8 years [18]. The above traditional methods were selected because they are rapid, simple, and effective techniques, and to our best knowledge, their comparison has never previously been performed.

## 2. Materials and Methods

The sample consisted of consecutive cases of children of French origin (Montpellier University Hospital) with the suspicion of child physical abuse/maltreatment. Inclusion criteria for the present study were: age of the subject equal or less than 8 years old (8 years of age was the upper limit of the sample), good oral hygiene, and absence of injury of the left hand and wrist. Exclusion criteria were: no known date of birth, dental trauma (loss, fracture, dislocation, extraction of teeth), dental pathology, orthodontic treatment, trauma/injury to the face or hand–wrist region and growth disorders/systemic illness. Finally, a total number of 94 cases were eligible; 64 males (68.1%) and 30 females (31.9%). For some individuals, the orientation of the X-rays did not permit the evaluation of Demirjian’s and Anderson’s stages.

X-ray surveys of the skull and left wrist and hand were collected in each case and analyzed by one skeletal (Greulich and Pyle atlas) and five dental age estimation methods (Anderson, Demirjian, Schour and Massler, and Ubelaker revised method, Gustafson and Koch). All the above examinations were taken in the course of diagnosis and treatment in cases of child physical abuse suspicion. 

All cases were numbered and the real age was blinded when age estimation was assessed by two experienced examiners using skeletal and dental methods. A forensic physician (LM) with fifteen years of experience performed bone age estimation and a forensic odontologist (CV) with ten years of experience performed dental age estimation. 

The differences between the chronological ages and estimated ages were calculated by the absolute difference between the chronological age and the estimated age. Inaccurancy is defined as the mean of the average absolute error. Bias and inaccuracy of each method were calculated. Inaccuracy is estimated as follows: |estimated age-chronological age|/(number of individuals). Bias is defined as follows: (estimated age-chronological age)/(number of individuals) and therefore indicates over or underestimation. Data were grouped into two categories, children with age equal or under 2 years old and children with age over 2 years old (*n* = 74, 78.7%, and *n* = 20, 21.3%, respectively). The above categorization of age was performed because the Anderson and Demirjian methods are applied only on the group of over 2-year-old subjects, as requested by the age limits of estimation of both methods. 

Statistical Agreement between age and the methods used to estimate age was assessed by intraclass correlation coefficient (ICC) and 95% confidence interval (CI). These dependent ICCs were compared via a likelihood ratio test [19]. The nonparametric Wilcoxon test for paired samples was used in order to compare the methods. Additionally, Bonferroni adjustments were applied for multiple comparisons. Statistical analyses were performed using SAS software (version 9.1). A *p*-value of less than 0.05 (two-tailed) was considered to indicate statistical significance.

Ethics approval was provided by Montpellier University Hospital, Programme Hospitalier de Recherche Clinique (PHRC) 2000, ref PROM 7634 in the context of a broader research program concerning child abuse. All the procedures performed in this study complied with the Declaration of Helsinki.

## 3. Results

**Figure 1:**
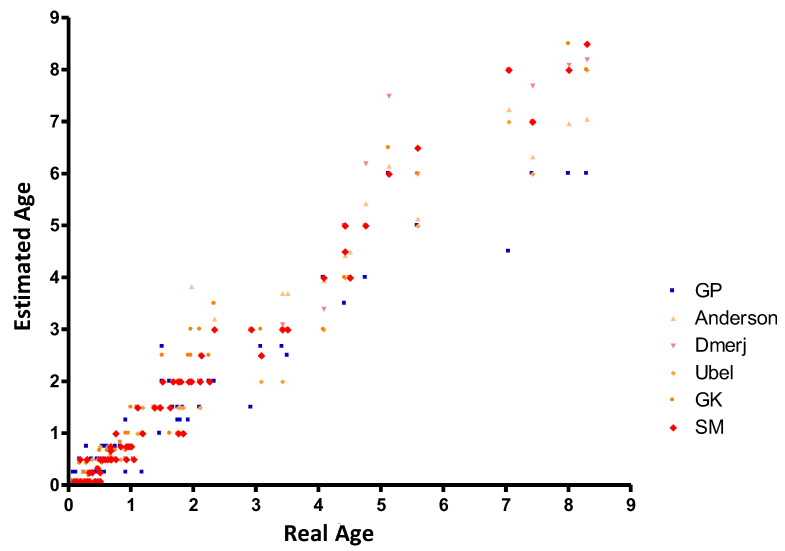
Real age and age estimated by the different methods. Notes. GP: Greulich and Pyle, GK: Gustafson and Koch, SM: Schour and Massler.

Descriptive statistics of real and estimated age via each method are presented in Table 1. ICC compared with chronological age for each method is shown in Table 2 and Figure 1. The highest ICC value was attained by the Gustafson and Koch method and Schour and Massler test (0.98), followed by the Ubelaker revision (0.97) and Greulich and Pyle atlas (0.94). The lowest ICC was attained by the Demirjian method (0.85). 

Biases under 2 years were similar in all methods, without significant difference between skeletal and odontological methods; over 2 years, all the odontological methods except Ubelaker’s one showed an overestimation, amounting to half a year for Demirjian. Greulich and Pyle’s atlas was the less precise, with an increasing underestimation in the age group over 2 years (Table 3).

Results concerning inaccuracy of skeletal and odontological methods in the two age groups (equal or under and over 2 years) are shown in Table 3. Application of the Greulich and Pyle atlas showed an increasing inaccuracy in children over 2 years, with a concurrent increase in deviation. Among dental methods, the Ubelaker revised method showed the lower inaccuracy for the age group ≤2 years, but results from both Gustafson and Koch and Schour and Massler are similar, with similar standard deviations. For the age group >2 years, inaccuracy of all the dental methods increased, but was always lower than that of the skeletal method. Among dental methods, the Demirjian was the least accurate, with a mean difference between the real and estimated age, amounting to 0.74 and the highest standard deviation among all the methods (Table 3). 

As the lowest inaccuracy in children under 2 years was found in Ublalker’s method, this method was compared with the other methods (Table 4). Only the Greulich and Pyle method gives significantly higher inaccuracy than the other methods (*p* = 0.035). Since the lowest inaccuracy in children older than two years was found in the method of Schour and Massler, this method was compared with the other methods (Table 4). Only the Greulich and Pyle method revealed significantly higher inaccuracy than other methods (*p* = 0.008). 

## 4. Discussion

Several methods for age estimation have been presented over the last 80 years based on the development of the teeth or skeleton. The dental age estimation is based either on the evaluation of the teeth in total or of a specific set (from one or both jaws or from one side), depending on the method used. They may also be divided into those using the atlas approach or those using scoring systems. The stage of tooth eruption or tooth formation may be used for the determination of dental maturity. The skeletal methods evaluate diverse skeletal maturity indicators, such as the ossification of bones of hand–wrist, epiphysis–diaphysis fusion, cervical vertebrae assessment, sternoclavicular bones assessment, changes in the pubic symphysis, and fusion of cranial sutures [13,14,15,16,17,20,21,22,23,24,25]. 

The selection of a method in a considered case depends on the available evidence. Dental methods have been argued as more reliable than skeletal ones, probably due to lesser influences by environmental factors and factors related to ancenstry [1,23,26]. However, the reliability of dental methods is not uniform from birth to adulthood. The combination of dental and skeletal methods has been suggested by several authors in order to achieve the best prediction of accuracy [2,9,23,27]. Cunha et al., in their review regarding aging human remains and living individuals, pointed out that a comparison between dental and skeletal age is always necessary to corroborate results and verify possible discrepancies [1]. Several previous studies have validated age estimation methods for different populations to test their effectiveness and accuracy [28,29,30]. However, variations in the age range of the sample and the ways that accuracy is reported make comparisons among them difficult. A further problem is that there is still no consensus among researchers regarding their application to all populations [26]. 

The aim of the present study was to compare five dental methods (Anderson, Demirjian, Schour and Massler, Ubelaker revised method, and Gustafson and Koch) and one skeletal (Greulich and Pyle atlas) (Table 5)in children of French origin belonging to the age group of 0 to 8 years old.

The Schour and Massler atlas is the first and most well-known one, which consists of a series of 21 drawings from the prenatal stage to adulthood [15]. The age categories are consecutive until the age of 12 years, and then a gap exists until the age of 15 years. The radiographic image of the entire maxillary and mandibular dentitions is compared to the above drawings. Rai et al. tested the applicability of the Schour and Massler atlas in children in India aged 5–15 years, divided into three groups. For the first group of 5–8-year-olds, the Schour and Massler method was found to underestimate the age in males (0.05 years) and overestimate the age in females (0.78 years) [31]. Cesário et al., in their study using the Schour and Massler atlas in a subadult Portuguese population aged 7–21 years old, found an underestimation of the chronological age with a mean difference of 5.4 months [32]. AlQahtani et al. assessed the accuracy of three dental methods, Schour and Massler, Ubelaker, and London Atlas, for consecutive age categories up to the age of 23.5 years. At birth, the mean difference between the dental and the chronological age was 0.09 years (overestimation), however, during the period from the age of 1.5 months up to 1.5 years, the use of the Schour and Massler atlas resulted in an underestimation ranging from 0.05 to 0.20 years. For the age groups from 2.5 years to 8.5 years old, a constant underestimation was observed with values ranging from 0.07 to 0.74 years (the minimum underestimation was observed at the age of 4.5 years). In our study, an underestimation with the Schour and Massler method amounting to 0.19 ± 0.16 years was found for the age group ≤ 2 years, but an overestimation of 0.40 ± 0.30 years for the age group > 2 years [33]. 

The Demirjian method was introduced in 1973 and it is probably the most widely used method for estimating the stage of developing teeth. It is a scoring system in which the development of the seven mandibular left permanent teeth is divided into eight stages, marked with alphabet letters from A to H [14]. The method assesses the central and lateral incisors, canines, first and second premolars, and first and second molars. The scoring system for the above method is differentiated between boys and girls and it is used to measure the maturation stage of teeth from 3 to 17 years of age. A meta-analysis by Jayaraman et al. involving 274 studies recorded over-estimation in most studies when using the Demirjian method [34]. Among the studies involved in the metanalysis, underestimation of the age was observed only in the Venezuelan subjects [35] and Western Chinese males [36] and accurate age estimation was demonstrated in a study conducted on Indian subjects [37]. The estimation results for the total population of the meta-analysis showed that the Demirjian method overestimated the age of females by 0.65 years and males by 0.60 years [34]. In a study on a Spanish population, the Demirjian method overestimated chronological age by 0.495 years on average for the age group under 14 years [38]. In a French study, Demirjian’s method was found to overestimate age by 1.17 years when applied to children aged 6.0–11.5 years old [39]. In addition, Yunus and Wardhani examined 30 panoramic radiograph samples of Indonesian children aged 4–9 years old using the Demirjian method [40]. The results showed low accuracy because of significant overestimation of dental age compared with the chronological age. An overestimation varying from 0.34 to 1.7 years was also reported in a retrospective study based on the evaluation of panoramic radiographs, belonging to 1006 children from Romania aged between 3 and 13.9 years [41]. Our results have also shown an overestimation of 0.74 ± 0.76 years in the age group > 2 years.

Gustafson and Koch combined anatomical, radiographic, and gingival eruption data to construct a chart of tooth formation and eruption for tooth types (excluding third molars) and age categories based on the radiographically observed development of the permanent teeth [17]. Application of the method in adopted in Sweden, non-European, children has shown a difference of a year or more [42]. In our study, underestimation was observed for the Gustafson and Koch method for both groups (age group ≤ 2 years with a mean difference 0.22 ± 0.23 and >2 years with a mean difference 0.53 ± 0.37). 

In the Anderson method, 14 mineralization stages have been determined for the maxillary and mandibular teeth of boys and girls [13]. Differences were observed between the two sexes, mainly regarding the canines. Variability in age was greater among the males and increased with age, except for the third molars. The standards of the Anderson method, although relatively easy and extensive, are problematic in that the original sample includes only ages more than two years [43]. In our study, an underestimation was also observed for the Anderson method for children older than 2 years old (mean difference 0.55 ± 0.45).

Ubelaker’s dental chart was loosely based on Schour and Massler’s atlas. Ubelaker drew from numerous data sources to correct the age range for each drawing. He included tooth emergence of North American Indians as “some studies suggest that teeth probably form and erupt earlier among Indians”. He altered the stages of tooth development and position within the diagrams of Schour and Massler’s atlas and introduced new additional ones. There was no differentiation in the data regarding the two sexes. Ubelaker revised his dental chart to accommodate the variations seen among individuals of the same chronological age [16]. Ubelaker’s atlas has been modified by Blenkin and Taylor, who reinterpeted data from previous studies in order to develop a more suitable, reliable, and simple tool for age estimation in the Australian population [44]. The accuracy of Ubelaker’s dental chart was assessed by AlQahtani et al., who found an underestimation for the age groups from 1.5 months to 7.5 years with a mean difference ranging from 0.00 to 0.59 years [33]. In concordance with the results of the previous study, in our study, the application of Ubelaker’s method resulted in underestimation of 0.17 ± 0.13 for children aged ≤ 2 years old and 0.56 ± 0.43 for children aged > 2 years old. Adams et al. tested Ubelaker’s atlas and the London Atlas [33] on a sample of 335 juvenile individuals, aged 5–25 years old, consisting of Native Americans, African Americans, European Americans, and New Mexico Hispanics. For Ubelaker’ s method, age estimation was recognized as correct if it corresponded to the age range provided by the method. According to the results of the above study, Ubelaker’s atlas showed higher accuracy, although lower precision, for estimating the chronological age of young people coming from different population backgrounds; probably because of the wider ranges provided for each age category. Regarding the age range from 5.00 to 7.99 years, the combination of both methods resulted in an overestimation of the chronological age ranging from 0.75 to 2.50 years. It is worth mentioning that the number of children involved in the study was small (*n* = 12) [45].

Examination of the hand–wrist is considered the easier and less time and expense-consuming skeletal method because it is facilitated by the absence of other hard tissues and the existence of several types of bones in the particular region [9,12]. Moreover, it includes many ossification centers in a small area [37]. Methods that evaluate the skeletal development of hand–wrist bones are those of Greulich and Pyle [18], Tanner–Whitehouse [46], and the FELS [47], with the first one being the most widely used. In our study, underestimation was observed for both age groups. The underestimation of results derived from the application of the Greulich and Pyle atlas is confirmed by several authors, who verified this information even in the youngest children (between 0 and 3 years) [48,49]. Data from the literature concerning this topic, however, are not univocal: the importance of environmental factors and factors related to ancestry, as well as the sex, should not be underestimated [6,50,51]. Ontell verified that the application of the Greulich and Pyle atlas usually underestimates females aged less than 8 years, and males less than 13 years; over this threshold, the use of the atlas tends to overestimate real age [52]. Hackman and Black, in their study on a Scottish population, found an overestimation for females 0–5 years old (2.25 months/0.19 years) and 6–10 years old (2.04 months/0.17 years), but an underestimation for males of the same age groups with a mean difference of 3.54 months/0.30 years and 2.44 months/0.20 years, respectively [53]. 

Statistical analysis of inaccuracy and bias in each method has a substantial contribution to the debate concerning the importance and limits of different age estimation methods; in our study, the dental and skeletal methods showed an excellent correlation with real age, especially under 2 years: in this age range, indeed inaccuracy from all the methods is similar, with a slight trend to underestimation. However, the most interesting result is the highest inaccuracy shown by the Schour and Massler and Ubelaker methods, based on diagrams, frequently used in the archaeological context, but usually considered less reliable than the statistical methods such as Demirjian’s. Even Gustafson and Koch’s method, based only on a verbal description of dental growth without the comparative illustration provided by more advanced methods such as Demirjian’s, proved to maintain high reliability in young children. There are no previous studies comparing the above methods on a given population and this information is undoubtedly hard to explain; probably in the age range between 0 and 8 years, dental development proceeds quickly, and this means that a higher number of dental modifications are visible than during adolescence or in juvenile adults. 

The rapid dental development during the above age period permits relatively easier recognition among the different ages, which seems easily achieved through the application of Schour and Massler and Unbelaker’s method, or the verbal description by Gustafson and Koch. In other words, the number of dental modifications does not require the application of more advanced procedures such as Demirjian’s, and diagram methods are reliable enough to reach a correct age estimation. Although the Demirjian chart has been validated in a large number of previous studies, the literature regarding the use of the other dental methods validated in the present study is limited. Olze et al. (2005) found that Gustafson and Koch’s method is affected by high inter- and intra-observer error ranges, whereas the Demirjian method proved to be the most reliable one. Their study, however, was performed with female German subjects aged 12–25 years old, a different population than the population of our study [54]. 

This study has several limitations. The first one is the lack of inter and intra observations; the age estimation for each method was assessed by a single investigator so there was not any ability to eliminate the variability that can arise from the human factor. Although this is the major limitation of the study, the authors believe that the systematic error associated with examiner reproducibility was reduced because the age estimation was performed by experienced experts. Two other limitations were the sample size, and specifically, the missing values that existed in some investigated methods and the age range (0–8 years). These two limitations are related to the sample on which the study is based.

## 5. Conclusions

The present study showed that, whereas for children aged ≤2 years, all the methods present similar inaccuracy, for children over 2 years, the diagram methods such as Schour and Massler and Ubelaker’s revised one have a lower error range than the most frequently used Greulich and Pyle atlas and Demirjian method. The above results have some interesting implications. From a general point of view, our results show that although literature suggests the use of statistical methods, which are supposed to be reliable and standardized, other methods should not be disregarded and that every method has a precise field of application within specific age ranges or in particular conditions. This study aims therefore at warning against the pure application of the most popular methods in forensics and points out the need for a wide study concerning the application of different procedures within the same population in order to know the in-depth potential and limits of each method.

## Figures and Tables

**Table 1:** Descriptive statistics of real age and age estimation for each method.

Variable	*n*	Mean	Standard Deviation	Max	Q75	Median	Q25	Min
Real age	94	1.49	1.83	8.29	1.83	0.678	0.33	0.08
Greulich Pyle (GP)	91	1.27	1.56	6.00	1.50	0.50	0.25	0.08
Anderson	14	5.12	1.41	7.24	6.33	4.82	3.83	3.21
Demirjian	9	6.47	1.99	8.20	8.00	7.50	6.00	3.10
Ubelaker	90	1.37	1.83	8.00	2.00	0.50	0.08	0.08
Gustafson-Koch (GK)	90	1.50	1.99	8.50	2.50	0.50	0.18	0.08
Schour Massler (SM)	90	1.45	1.99	8.50	2.00	0.50	0.08	0.08

**Table 2:** Intraclass correlation coefficients (ICC) with 95% confidence intervals (CI).

Methods	ICC Compared with Real Age	Bonferroni-Adjusted *p*-Value (Compared with ICC between Age and SM Method)
Greulich and Pyle	0.94 (0.91–0.96)	<0.001
Anderson	0.88 (0.68–0.96)	<0.001
Demirjian	0.85 (0.50–0.96)	N/A
Ubelaker	0.97 (0.97–0.99)	0.16
Gustafson and Koch (GK)	0.98 (0.97–0.98)	0.06
Schour and Massler (SM)	0.98 (0.98–0.99)	-

Notes. In Demirjian method, *p*-value could not be computed due to missing values.

**Table 3:** Mean (and standard deviation) of bias and inaccuracy under and over 2 years.

	Bias (in Years)				Inaccuracy (in Years)			
	≤2 Years		>2 Years		≤2 Years		>2 Years	
	*n*	M ± SD	*n*	M ± SD	*n*	M ± SD	*n*	M ± SD
Greulich and Pyle	70	−0.09 ± 0.31	20	−0.73 ± 0.87	70	0.24 ± 0.21	20	0.88 ± 0.72
Anderson	N/A		13	−0.05 ± 0.73	N/A		13	0.55 ± 0.45
Demirjian	N/A		9	0.50 ± 0.73	N/A		9	0.74 ± 0.76
Ubelaker	70	−0.09 ± 0.20	20	−0.27 ± 0.66	70	0.17 ± 0.13	20	0.56 ± 0.43
Gustafson and Koch	70	−0.06 ± 0.31	20	0.15 ± 0.64	70	0.22 ± 0.23	20	0.53 ± 0.37
Schour and Massler	70	−0.11 ± 0.23	20	0.10 ± 0.50	70	0.19 ± 0.16	20	0.40 ± 0.30

Notes. M: mean, SD: standard deviation.

**Table 4:** Comparison of inaccuracy between methods of age evaluation.

Children under or Egal to 2 Years		Children over 2 Years	
Ubelaker vs. Greulich and Pyle	*p* = 0.035	Schour and Massler vs. Greulich and Pyle	*p* = 0.008
Ubelaker vs. Gustafson and Koch	*p* = 0.245	Schour and Massler vs. Anderson	*p* = 0.685
Ubelaker vs. Schour and Massler	*p* = 0.432	Schour and Massler vs. Dermijian	*p* = 0.624
		Schour and Massler vs. Ubelaker	*p* = 0.108
		Schour and Massler vs. Gustafson and Koch	*p* = 0.148

**Table 5:** Description of the methods used in the study.

Methods	Brief Description	Pédiod of Use
Greulich and Pyle (1959)	Carpal and phalangeal epiphyses stage of ossification of left hand and wrist compare to atlas	First year of live to 19
Schour and Massler (1937)	Comparative analysis of orthopantomograms (OPGs) and images available in the corresponding chart	From in utero to aldulthood
Demirjian (1973)	Scoring system in which the development of the seven mandibular left permanent teeth is divided into eight stages (A to H)	Three to 17 years of age
Gustafson and Koch (1974)	Comparative analysis on OPGs of diagrams of four stage of tooth development	First year of life to 16
Anderson (1976)	Comparative analysis on OPGs of 14 stages of mineralization	Four to 14 years of age
Ubelaker (1978)	Comparative analysis of OPGs and images available in the corresponding chart	From in utero to aldulthood

## Data Availability

All data generated or analyzed during this study are included in this published article or are available from the corresponding author on reasonable request.
